# MSN在多发性骨髓瘤中的临床意义及致病机制研究

**DOI:** 10.3760/cma.j.issn.0253-2727.2023.08.010

**Published:** 2023-08

**Authors:** 雨晴 高, 春丽 徐, 宏煜 付, 婷婷 朱, 剑虹 储

**Affiliations:** 1 苏州大学苏州医学院造血干细胞移植研究所，国家血液系统疾病临床医学研究中心，苏州大学附属第一医院，江苏省血液研究所，血液学协同创新中心，苏州 215000 Institute of Blood and Marrow Transplantation, Medical College of Soochow University, National Clinical Research Center for Hematologic Diseases, The First Affiliated Hospital of Soochow University, Jiangsu Institute of Hematology, Collaborative Innovation Center of Hematology, Suzhou 215000, China; 2 苏州大学附属儿童医院，苏州 215000 Children's Hospital of Soochow University, Suzhou 215000, China; 3 东台市人民医院检验科，东台 224200 Department of Clinical Laboratory, Dongtai Municipal People's Hospital, Dongtai 224200, China

多发性骨髓瘤（MM）是浆细胞的血液学恶性肿瘤，其特征是单克隆免疫球蛋白的过度增殖，好发于中老年人[1]–[2]。尽管现有的治疗方法使MM治疗的部分缓解率和完全缓解率有所提高，但高危患者的治疗难度大、预后差[3]–[4]。探寻新的MM治疗靶点可为MM提供新的治疗策略。MSN是一种细胞骨架蛋白，主要在内皮细胞中表达，在细胞增殖、迁移和黏附中发挥重要作用[5]–[7]。在本研究中，我们通过体外实验初步验证了MSN在MM中的致病机制。

## 材料与方法

1. 试剂与仪器：ACEA NovoCyte流式细胞仪购自艾森生物（杭州）有限公司，TRIzol总RNA抽提试剂、HiScript^®^ Ⅲ 1st Strand cDNA合成试剂盒和NovoStart^®^ SYBR qPCR SuperMix Plus试剂盒均购自南京诺唯赞生物科技有限公司，RIPA蛋白裂解液和BCA蛋白定量试剂盒均购自上海碧云天生物技术有限公司，Tanon 5200全自动化学发光成像分析系统和ECL化学发光试剂盒均购自上海天能科技有限公司，CCK8试剂盒购自美国Bimake公司，SYBR green荧光染料购自美国Proteintech公司，DMEM和RPMI 1640培养基购自美国Gibco公司。兔抗人MSN（3146S），兔抗人PARP（9542S）以及兔抗人p-H2A.X（9718S）购自美国CST公司，鼠抗人β-actin（ab008-040）以及辣根过氧化物酶（HRP）标记山羊抗兔、鼠均购自杭州联科技术股份有限公司，实验相关引物由苏州金唯智生物科技有限公司合成。

2. 细胞培养：人MM细胞系RPMI-8226、MM.1S、MM.1R、U266和NCI-H929购自南京科佰生物科技有限公司或中国科学院细胞库，培养条件：RPMI 1640培养基（10％胎牛血清、青霉素和链霉素各100 µg/ml），恒温培养箱（37 °C、5％ CO_2_、95％湿度）中培养，每2～3 d换液或传代，由本实验室对细胞进行培养。

3. 实时荧光定量PCR检测MSN mRNA表达水平：使用TRIzol法提取细胞总RNA，并用HiScript^®^ Ⅲ 1st Strand cDNA试剂盒将RNA逆转录成cDNA，使用NovoStart^®^ SYBR qPCR SuperMix Plus试剂盒进行实时荧光定量检测，以β-actin为内参，以2^−∆∆Ct^法计算出MSN基因的相对表达量。

4. 使用慢病毒构建敲低MSN的细胞株：敲低载体为pLKO.1-TRC-GFP，酶切位点选择Xba Ⅰ和Age Ⅰ，将shMSN#2、shMSN#3的shRNA干扰序列退火后的退火产物与经Xba Ⅰ和Age Ⅰ酶切后的载体进行连接反应，构建相应干扰质粒，并将空载体shscramble作为对照组，选择状态良好的HEK-293T细胞包装慢病毒，48 h后收集病毒上清感染MM.1S和NCI-H929细胞株，感染72 h后使用流式细胞仪检测GFP阳性的细胞占全部细胞的比例，以反映慢病毒的感染效率，使用荧光定量PCR和Western blot技术分别在mRNA和蛋白水平上检测敲低MSN的效率。

5. Western blot检测MSN、PARP、p-H2A.X蛋白表达水平：使用RIPA蛋白裂解液制备细胞总蛋白并使用BCA 蛋白定量试剂盒测定蛋白浓度。每孔使用等量蛋白进行SDS-PAGE电泳，然后将分离的蛋白条带转移至0.45 µm的PVDF 膜上，5％脱脂牛奶封闭后，一抗4 °C孵育过夜，二抗室温孵育1 h。使用ECL化学发光试剂盒处理PVDF膜，Tanon全自动化学发光成像分析系统成像。

6. CCK-8法检测细胞增殖能力：以转染后72 h为时间起点，将MM.1S和NCI-H929细胞按每孔3×10^3^个接种到96孔板中，每孔体积100 µl，利用CCK-8试剂检测0、2、4、6 d 4个时间点的细胞增殖能力，对应时间段每孔加入10 µl CCK8试剂，每个时间点在450 nm处的吸光度（*A*_450_）表示细胞的增殖能力，实验重复3次。

7. 集落形成实验：将0.66％的琼脂糖液和2×RMPI-1640（加入2×抗生素和20％的胎牛血清）等比例混合，细胞离心计数，每孔种3 000个细胞，将细胞与混合液轻柔混匀后，取3 ml加入6孔板中。培养箱中培养2～3周后，结晶紫染色，计数克隆形成数量。

8. Annexin V-APC/7-AAD染色法检测细胞凋亡水平：收集对照组、shMSN#2、shMSN#3的MM.1S和NCI-H929细胞，800×*g*离心5 min，弃上清。按细胞凋亡试剂盒说明书操作加入Annexin V-APC/7-AAD抗体，室温避光染色15 min后用流式细胞仪检测细胞凋亡。以早期和晚期凋亡细胞占总细胞数的比值计算细胞凋亡率，实验重复3次。

9. 统计学处理：使用GraphPad Prism 9.0进行统计学分析和制图。计量资料符合正态分布的采用*t*检验，不符合正态分布的采用秩和检验，率的比较采用卡方检验，相关性采用皮尔森相关系数表示，*P*<0.05表示差异具有统计学意义。

## 结果

1. MSN在MM细胞系中的表达：检测MM细胞RPMI-8226、MM.1S、MM.1R、U266和NCI-H929细胞中MSN mRNA和蛋白表达水平，其中U266、MM.1S、MM.1R和NCI-H929细胞MSN mRNA和蛋白水平偏高，RPMI-8226的表达水平偏低（图1），因此选取NCI-H929和MM.1S细胞作为研究MSN在MM中的生物学功能的工具细胞。

**图1:**
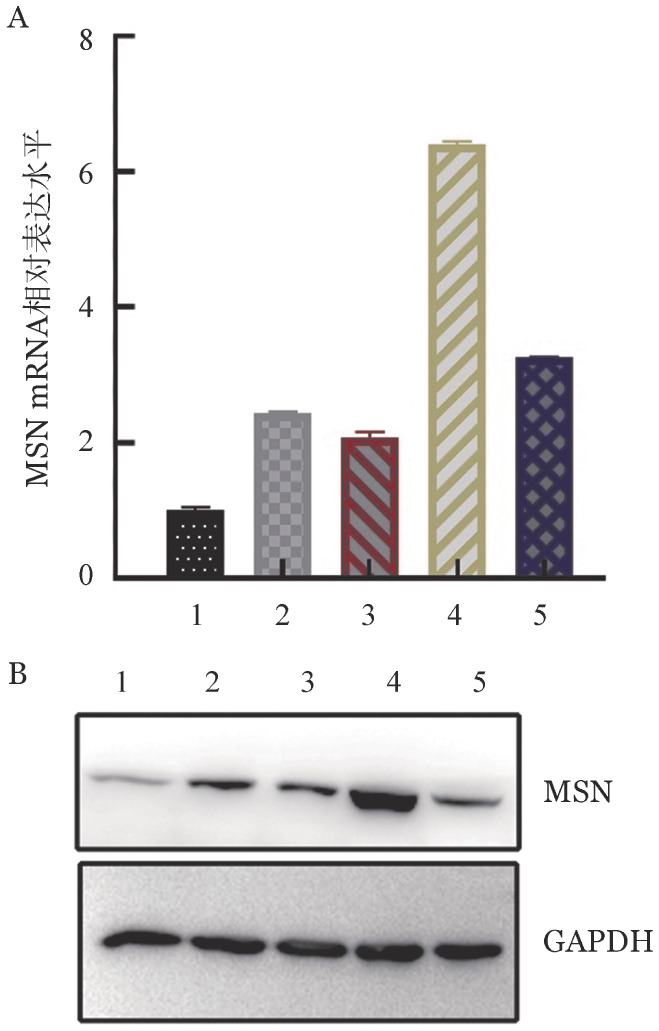
5种多发性骨髓瘤细胞株中MSN mRNA（A）和蛋白（B）的表达水平 注 1：RPMI-8226细胞；2：MM.1S细胞；3：MM.1R细胞；4：U266细胞；5：NCI-H929细胞

2. 敲低MSN的MM细胞系的建立：慢病毒转染72 h后，感染效率均在90％以上。与对照组相比，shMSN#2和shMSN#3中MM.1S细胞和NCI-H929细胞MSN的mRNA和蛋白水平均显著下调（图2），证明利用shRNA技术在MM细胞系中能成功敲低MSN的表达。

**图2:**
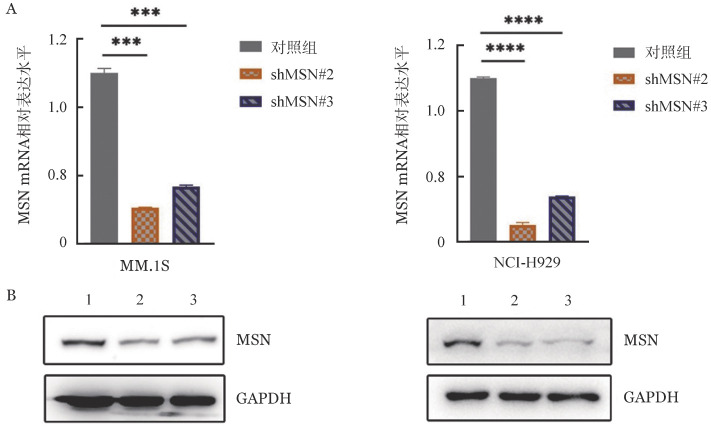
敲低MSN后MM.1S和NCI-H929细胞中MSN mRNA（A）和蛋白（B）表达水平的变化（****P<0.0001，***P<0.001） 1：对照组；2：shMSN#2组；3：shMSN#3组

3. 敲低MSN对MM细胞增殖能力的影响：敲低MSN后MM.1S和NCI-H929细胞增殖均受到显著抑制（*P*<0.01和*P*<0.0001）（图3）。敲低MSN后MM.1S细胞中，对照组克隆数为（184±24）个，shMSN#2和shMSN#3克隆数分别为（76±9）个，（40±6）个；敲低MSN后NCI-H929细胞中，对照组克隆数为（145±13）个，shMSN#2和shMSN#3克隆数分别为（38±1）个，（2±1）个。与对照组相比，敲低MSN后 MM.1S和NCI-H929细胞的集落形成能力明显减弱（*P*<0.05，*P*<0.01）（图4）。

**图3:**
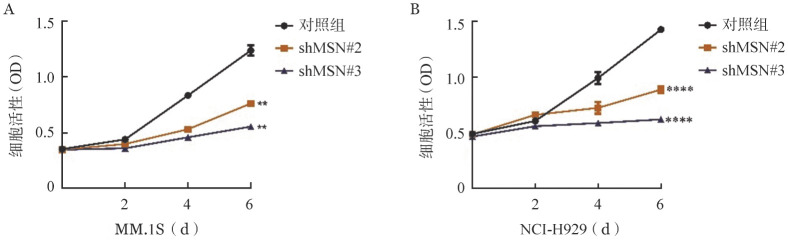
CCK-8检测MSN敲低后MM.1S和NCI-H929细胞中对细胞增殖能力的影响（与对照组相比，****P<0.0001，**P<0.01）

**图4:**
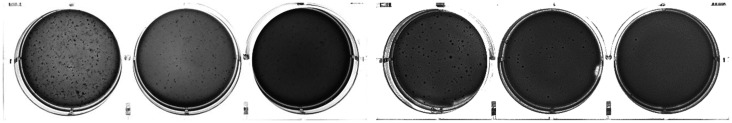
软琼脂集落形成实验检测敲低MSN后MM.1S和NCI-H929细胞中集落形成能力变化

4. 敲低MSN对MM细胞凋亡的影响：MM.1S细胞中，对照组凋亡率为（11.06±0.29）％，shMSN#2和shMSN#3凋亡率分别为（32.97±0.93）％，（70.5±1.51）％；NCI-H929细胞中，对照组凋亡率为（13.75±4.73）％，shMSN#2和shMSN#3凋亡率分别为（24.58±1.89）％，（40.46±4.57）％。与对照组相比，敲低MSN后MM.1S和NCI-H929细胞凋亡率均显著增加（*P*<0.05）。

5. 敲低MSN对MM细胞中的DNA损伤的影响：与对照组相比，shMSN#2和shMSN#3中MM.1S和NCI-H929细胞Cleaved-PARP表达水平增加，同时p-H2A.X蛋白表达量升高（图5）。

**图5:**
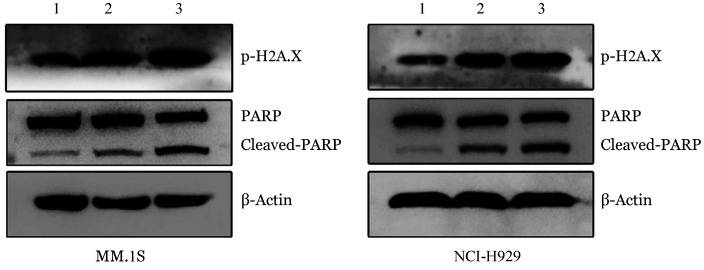
Western blot法检测敲低MSN后MM.1S和NCI-H929细胞中Cleaved-PARP和p-H2A.X表达水平 1：对照组；2：shMSN#2组；3：shMSN#3组

## 讨论

耐药和复发是治疗MM的主要难题，基因组不稳定性和异常DNA损伤修复与耐药性的发展和疾病进展有关，是癌细胞的显著特征[9]–[10]。MSN是ezrin-radixin-moesin（ERM）家族的成员，定位在X染色体，MSN由三个部分组成：一个与质膜结合的N端FERM域，一个C端为肌动蛋白结合位点以及连接N端和C端的α螺旋区[11]–[12]。在造血过程中，MSN和ezrin都表达，而radixin大部分缺失，MSN也是ERM家族中唯一在血小板表达的成员[13]。正常情况下，MSN主要参与维持并调节细胞的正常生理水平等过程；但是在病理情况下，MSN在多种肿瘤中高表达，并参与肿瘤的发生发展，与肿瘤的预后密切相关。越来越多的证据表明，MSN表达及其亚细胞定位改变导致的细胞信号调节失调在口腔鳞状细胞癌、黑色素瘤和乳腺癌[14]–[16]。MSN已成为许多癌症的治疗靶点。然而，迄今为止，关于MSN的研究主要局限于实体瘤，血液肿瘤研究不多，参与肿瘤的发生发展过程中作用的机制也不够深入。本课题通过探讨MSN在MM中的临床意义发现MSN的表达水平在MM患者中较高，其中，肿瘤分期Ⅲ期MSN表达水平较Ⅰ、Ⅱ期高，这提示我们MSN高表达与MM发生发展相关，但两者发生机制仍需要进一步研究。本研究通过体外功能实验，发现敲低MSN后MM细胞增殖显著抑制，凋亡率显著增加，提示MSN可能通过促进MM细胞增殖从而促进疾病侵袭性增加、耐药等恶性病程。本研究进一步Western blot检测发现敲低MSN后PARP蛋白剪切体形式增加，p-H2A.X蛋白表达量升高，DNA损伤后发生的最早事件之一是组蛋白H2A.X上Ser139的磷酸化，p-H2AX是DNA双链损伤的标志蛋白，参与DNA损伤应答（DDR）[17]，提示敲低MSN可引起MM细胞发生不可修复的DNA损伤从而导致细胞凋亡。

综上所述，MSN在MM中高表达且参与MM的发生发展，并通过引起DNA损伤细胞反应从而影响MM细胞增殖和活力，但是MSN在MM中诱导DDR反应的具体机制，仍需进一步研究。随着研究深入MSN可能被认为是MM临床治疗一种新的有前景的靶点。
